# Effect of a *Euglena gracilis* Fermentate on Immune Function in Healthy, Active Adults: A Randomized, Double-Blind, Placebo-Controlled Trial

**DOI:** 10.3390/nu11122926

**Published:** 2019-12-03

**Authors:** Malkanthi Evans, Paul H. Falcone, David C. Crowley, Abdul M. Sulley, Marybelle Campbell, Nisrine Zakaria, Joanne A. Lasrado, Emily Pankow Fritz, Kelli A. Herrlinger

**Affiliations:** 1 KGK Science, London, ON N6A 5R8, Canada; 2 Kemin Foods LC, Des Moines, IA 50317, USA

**Keywords:** beta-glucan, algae, immune, upper respiratory tract infection, exercise

## Abstract

*Euglena gracilis* produce high amounts of algal β-1,3-glucan, which evoke an immune response when consumed. This study investigated the effect of supplementation with a proprietary *Euglena gracilis* fermentate (BG), containing greater than 50% β-1,3-glucan, on immune function as measured by self-reported changes in upper respiratory tract infection (URTI) symptoms. Thirty-four healthy, endurance-trained participants were randomized and received either 367 mg of BG or placebo (PLA) for 90 days. Symptoms were assessed by the 24-item Wisconsin Upper Respiratory Symptom Survey and safety via clinical chemistry, hematology, vitals, and adverse event reporting. Participants supplemented with BG over 90 days reported fewer sick days (BG: 1.46 ± 1.01; PLA: 4.79 ± 1.47 days; *p* = 0.041), fewer URTI symptoms (BG: 12.62 ± 5.92; PLA: 42.29 ± 13.17; *p* = 0.029), fewer symptom days (BG: 5.46 ± 1.89; PLA: 15.43 ± 4.59 days; *p* = 0.019), fewer episodes (BG: 2.62 ± 0.67; PLA: 4.79 ± 0.67; *p* = 0.032), and lower global severity measured as area under curve for URTI symptoms (BG: 17.50 ± 8.41; PLA: 89.79 ± 38.92; *p* = 0.0499) per person compared to placebo. Sick days, symptoms, and global severity were significantly (*p* < 0.05) fewer over 30 days in the BG group compared to PLA. All safety outcomes were within clinically normal ranges. The study provides evidence that supplementation with a proprietary *Euglena gracilis* fermentate containing greater than 50% β-1,3-glucan may reduce and prevent URTI symptoms, providing immune support and protecting overall health.

## 1. Introduction

Upper respiratory tract infections (URTI) cause cold- and flu-like symptoms and are the most common form of acute illness with roughly 70% of the general population experiencing at least one episode annually [1,2,3,4,5]. Lost productivity due to cold- and flu-like symptoms has a drastic impact on the economy with an estimated 40 million missed work or school days each year, translating to over $40 billion in lost economic potential [6]. Supplementation with naturally occurring bioactive molecules and functional foods can have a beneficial effect on immune function [7] and it may be a potential strategy for preventing productivity losses due to colds.

*Euglena gracilis* is a micro-algae of the division Euglenophyceae and family Euglenales [8]. Classified as a protist, *Euglena gracilis* can synthesize many nutrients under autotrophic and heterotrophic growth conditions, including protein, omega-3 fatty acids, vitamin C, vitamin E, and other anti-oxidants [9]. Omega-3 fatty acids can regulate both innate and adaptive immunity [7] and vitamin C and E supplementation can enhance immune function by increasing neutrophil chemotaxis and phagocytosis and by modulating T cell function, respectively [10,11]. Importantly, *E. gracilis* produces paramylon, a high molecular weight, linear, unbranched, insoluble β-1,3-glucan, which acts as energy storage within the cell. Previous research has linked insoluble β-1,3-glucans with human immunity. β-1,3-glucans carry non-self, pathogen-associated molecular patterns (PAMPs) that are non-specifically recognized by cell surface receptors in the body, prompting an innate immune response [12]. Consequently, supplementation with *E. gracilis* has the potential to support immune function [12,13,14].

Monitoring the variable incidence of URTI symptoms in a healthy population is challenging and requires that participants are followed for a sufficient duration, thereby presuming an eventual occurrence of a URTI. Certain stressors, however, such as exercise, lifestyle, and psychological stress, can negatively affect the immune response. These stressors provide an opportunity to verify immune support benefits of interventions in healthy individuals. Previous studies have recruited populations such as healthcare providers or firefighters [15,16]. Additionally, active individuals participating in endurance training at higher levels of intensity and duration experience higher rates of cold- and flu-like symptoms than the general population [17]. The increased prevalence of cold- and flu-like symptoms is attributed to a hormetic relationship between exercise and immune function, with higher levels of exercise leading to transient immunosuppression [18,19]. Exercise-induced immunosuppression can last from 24 h [20,21] to 2 weeks [22] following training sessions or endurance events. The disruption in immunity caused by endurance exercise may be minimized by immunonutrition [19]. Therefore, endurance-trained individuals may provide an ideal population to study the effects of a supplementation regimen intended to strengthen the immune system in healthy individuals. The purpose of this study was to investigate the potential immunomodulating effects of a proprietary *Euglena gracilis* fermentate (BG) containing greater than 50% β-1,3-glucan measured by self-reported changes in URTI symptoms in healthy, active individuals at greater risk for URTI over 90 days compared to a placebo.

## 2. Materials and Methods

The study was approved by the institutional review board (IRB Services, Aurora, Ontario, Canada) on 5 January 2018 (Pro00023994) and by Health Canada (#233692). The trial was performed according to the ethical guidelines detailed in the Declaration of Helsinki (2008) and complied with the International Council for Harmonization of Technical Requirements for Pharmaceuticals for Human Use (ICH) guidelines. Researchers strictly followed the ICH guidelines for Good Clinical Practice Current Step 4 Version, dated 10 June 1996. The researchers also ensured the archiving of essential documents. The present study was registered at clinicaltrials.gov (NCT03518281) prior to recruitment and followed the CONSORT guidelines for randomized controlled trials [23] (Supplementary Table S1). After the study was explained verbally, written information was provided, written consent was obtained, and a copy of the consent was provided to the participant.

### 2.1. Investigational Product

The *Euglena gracilis* fermentate (Commercial name BetaVia^TM^ Complete, Kemin Foods LC, Des Moines, IA, USA) was produced from the *Euglena gracilis Kelbs* var. *bacillaris* ATCC PTA-123017 strain. The 1,3-β-glucan content in BetaVia Complete was quantified by compendial methods for total dietary fiber (TDF) according to the AOAC Official Method 991.43 Total, Soluble, and Insoluble Dietary Fiber in Foods.

Nuclear magnetic resonance spectroscopy was used for structural analysis of β-glucans, providing information on the glycosidic linkages. The acquired spectra for BetaVia Complete precipitate fractions agree by chemical shift, integration, and coupling constant with a commercially acquired paramylon sample and literature reports for 1,3-β-glucan [24,25].

Genotoxocity and subchronic toxicity studies of the dried fermentate from the same proprietary strain of *E. gracilis* found no evidence of toxicity up to the limit of 50,000 ppm from diet. The authors also found no mutagenic properties and there were no signs of gross toxicity, adverse effects, or abnormal behavior as the rats appeared active and healthy throughout the study. In addition, BG has widespread use in conventional food products, which have been regulated for food-grade specifications and BetaVia Complete has been affirmed as Generally Recognized as Safe for use in a variety of food and beverage applications. As such, it was deemed safe for human consumption [26].

### 2.2. Study Design

The present study was a 90-day single-center, randomized, double-blind, placebo-controlled, parallel-group trial conducted at the KGK Science Inc. clinical trial center in London, Ontario, Canada from 19 January 2018 to 10 August 2018. Participants were asked to consume one capsule containing 367 mg of BG or placebo (PLA; microcrystalline cellulose) once daily 30 min before breakfast for a duration of 90 days. There were no differences in size, color, taste, or texture between BG and PLA.

### 2.3. Study Population

Generally healthy, endurance-trained (1.5–3 h/day of endurance exercise or sport, 5–6 days/week) men and women between 21 and 65 years of age with a body mass index ranging from 18 to 35 kg/m^2^ who were willing to maintain a consistent diet and lifestyle (including exercise) routine were included in the study. At the initial screening visit, participants underwent a physical examination by a physician including routine hematology, clinical chemistry, and vitals to confirm health status. Inclusion and exclusion criteria are detailed in Table 1. As per the original protocol, flu vaccination was an exclusion criterion and individuals that had received the flu vaccine were not enrolled into the study. Based on the 2017–2018 data, the flu vaccine was reported to be only 10–20% effective against the major viral strain in Canada (H3N2). Therefore, it was expected that the vaccine may not have a major impact on the study outcomes and individuals who received the vaccine could participate in the study. A mid-study amendment was made to remove the flu vaccination as an exclusion criterion.

### 2.4. Study Procedure

**Figure 1:**
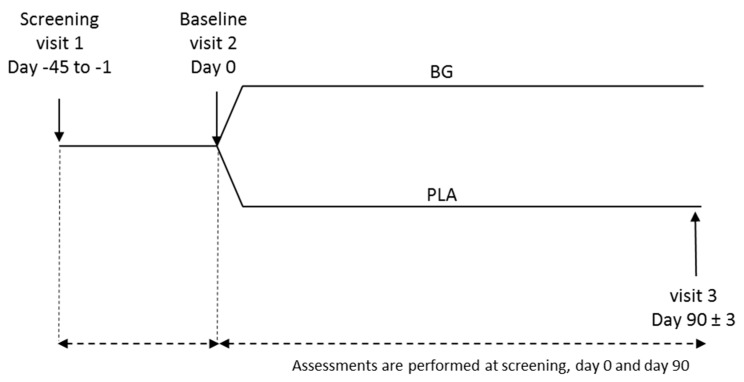
Study flow diagram.

The schematic depiction of the study design is shown in Figure 1. The study included three visits to the clinical trial center: screening (Visit 1), baseline (Visit 2), and post-supplementation (Visit 3). Safety parameters were measured at clinic visits, while efficacy was determined via daily administration of the Wisconsin Upper Respiratory Symptom Survey-24 (WURSS-24) during the 90-day supplementation period. At Visit 1, potential participants were screened via medical history, inclusion/exclusion criteria, concomitant therapies, and current health status. At Visit 2 (baseline), participants were randomized into either BG or PLA groups. After 90 days of supplementation, participants returned for Visit 3, where efficacy and compliance were assessed, and safety was measured.

A block randomization list was generated by unblinded personnel at KGK Science Inc. who were not involved in any data collection. Block randomization with a block size of 4 was implemented, such that two participants each were allocated to BG and PLA groups within each block in a random fashion. A randomization schedule that matched sequentially numbered containers to potential participants (who were assigned separate randomization numbers at baseline) was provided to the investigator, and all participants and site personnel involved in data collection were blinded to the product. Emergency unblinding envelopes were prepared for each participant. This randomization process allowed for the possibility of unblinding individual participants—in case of emergency—without unblinding the entire study. The study was unblinded after data collection was completed and initial study reports with statistical analyses were prepared.

### 2.5. Wisconsin Upper Respiratory Symptom Survey-24 (WURSS-24)

The validated WURSS-24 questionnaire was administered daily to assess URTI incidence, duration, and severity [1]. It consists of 2 general URTI questions, 13 symptom-related questions, and 9 quality of life or daily functioning questions. The responses are rated from 0 (not sick or no symptom) to 7 (severely or severe), with a maximum total score of 91 and 63 for symptom-related and daily-functioning questions, respectively. A higher score is indicative of a more severe cold and a greater number of cold symptoms. The cold symptoms assessed included runny nose, plugged nose, sneezing, sore throat, scratchy throat, cough, hoarseness, head congestion, chest congestion, feeling tired, headache, body aches, and fever.

### 2.6. Outcomes

In the present study, the primary outcome was the percentage of participants with very mild, mild, moderate, or severe URTI symptoms in the BG group and the PLA group after 30 or 90 days of supplementation.

Secondary outcomes included: (1) number of sick days per person (defined as days scored as “sick”); (2) the total number of URTI symptoms experienced per person; (3) number of days with URTI symptoms per person; (4) number of URTI episodes per person (defined as the appearance of one or more symptoms with at least two days of “not sick” in between); and (5) global severity of symptoms per person determined by the mean area under the curve (AUC) for WURSS-24 daily symptom scores during 30 or 90 days of supplementation with BG or PLA [27,28].

### 2.7. Safety

Safety measurements included hematology, clinical chemistry, and vital signs. Pre- and post-supplementation blood samples were collected via venipuncture by a phlebotomist and analyzed for complete blood count and clinical chemistry (sodium, potassium, chloride, calcium, creatinine, estimated glomerular filtration rate (eGFR), aspartate aminotransferase (AST), alanine aminotransferase (ALT), and bilirubin). Vital signs (systolic and diastolic blood pressure and heart rate) were measured via an automated digital sphygmomanometer (American Diagnostic Corporation Model No. 6022). In addition to the above, participants were queried on adverse events (AEs) throughout the trial.

### 2.8. Statistical Analyses

Power calculations were performed using the test of two proportions to determine the required sample size to provide 80% power at the 0.05 alpha level based on the primary outcome, which was a comparison of the percentage of participants with URTI symptoms between the BG group and the PLA group. In total, 60 participants were required at enrollment, based on an estimated 20% attrition over the course of this study, to detect a 45% difference in the incidence of URTI symptoms between groups [29].

All analyses were performed by an independent, third-party statistician using SAS for Windows version 9.3 (SAS Institute Inc, Cary, NC, USA); a two-tailed *p* value of <0.05 was considered statistically significant. All data are presented as mean ± standard error of the mean (SEM). Between-group differences for the primary outcome, demographics, and adverse events were assessed by the chi-squared or Fisher’s exact test, where appropriate. All secondary outcomes were assessed by ANOVA, wherein the data collected from Day 1 to Day 30 and Day 1 to Day 90 were compared between groups. Safety outcomes were assessed by ANOVA and compared from screening to Day 90 between groups.

The safety population consisted of all participants who received capsules and with any available post-randomization efficacy information. The per protocol (PP) population included all participants who consumed at least 80% of either study product, did not have any major protocol violations, and completed all study visits and procedures connected with measurement of the primary variables. Efficacy outcomes were analyzed in the PP population while safety outcomes were analyzed in the safety population.

Post-hoc analyses, not initially described in the statistical analysis plan, were conducted on individual symptoms and total overall (symptom plus functional) outcomes by ANOVA. The statistician remained blinded throughout the process of analyzing data.

## 3. Results

**Figure 2:**
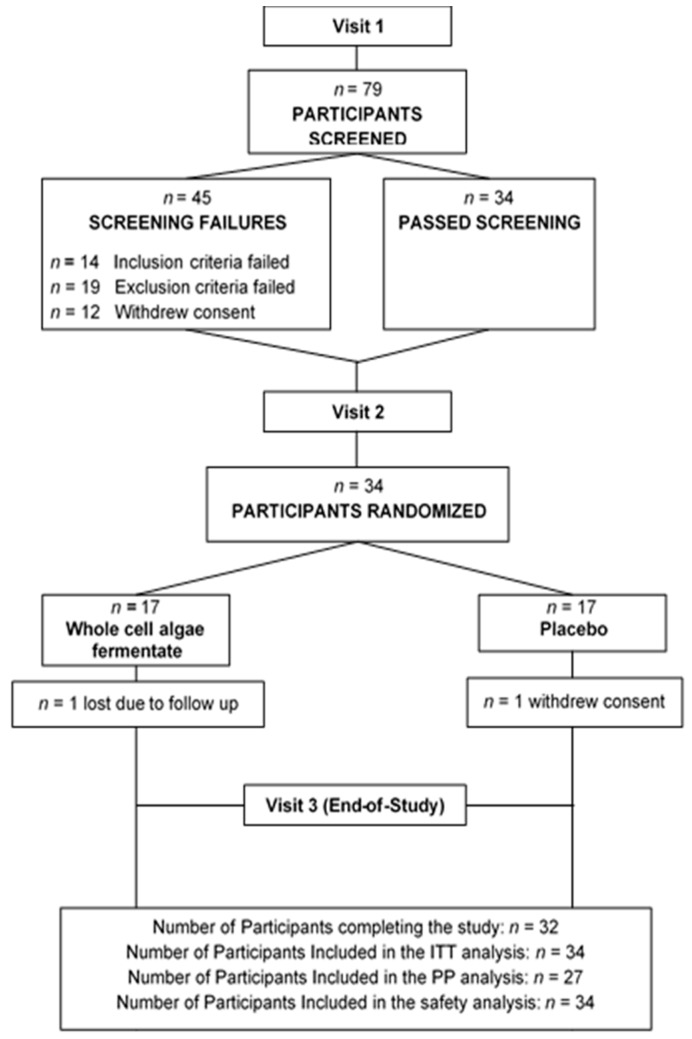
Disposition of study participants. From the whole cell algae fermentate group, 3 participants were removed from the PP population, 1 for non-compliance and 2 for missing >10% of primary endpoint data. From the placebo group,2 participants were removed from the PP population, 1 for non-compliance and 1 for immunomodulatory use.

A total of 79 participants were screened and 34 eligible participants were enrolled in the study with 17 participants each in the BG and PLA groups (Figure 2). Thirty-two participants completed the study, and five participants were removed from the per protocol (PP) analysis for non-compliance regarding product, data reporting, or exclusionary medication, resulting in a PP of 13 and 14 in the BG and PLA groups, respectively. Recruitment into the study was terminated early to ensure data collection during a single cold and flu season—as verified by incidence rates by the Middlesex London Health Unit (London, Ontario) [30]—thereby limiting the impact of seasonal variation of exercise and dietary behaviors in endurance-trained individuals. The demographic details of the participants are provided (Table 2). Both groups were well-balanced with no significant differences observed in age, sex, or ethnicity between groups. Product compliance was 98.90% and 100.00% in the BG and PLA groups, respectively.

Regarding the primary outcome, the percentages of participants reporting URTI symptoms are detailed in Table 3. Between-group differences for very mild, mild, moderate, severe, or any symptoms were not statistically significant, although a trend was observed in very mild symptoms from Day 1 to Day 90 (*p* = 0.098).

**Figure 3:**
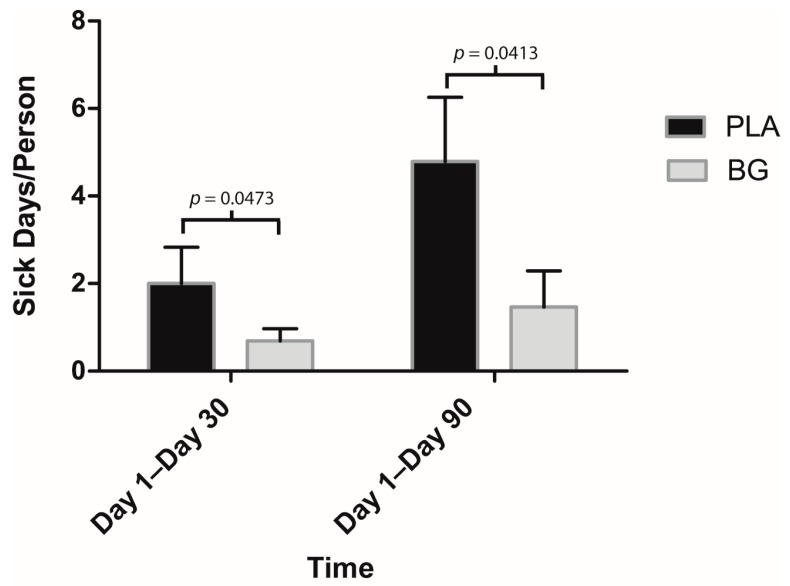
Mean number of sick days per person after 30 and 90 days of supplementation with BG or PLA in the PP population (*n* = 27). Values are mean ± SEM. BG, whole cell algae fermentate; PLA, placebo.

**Figure 4:**
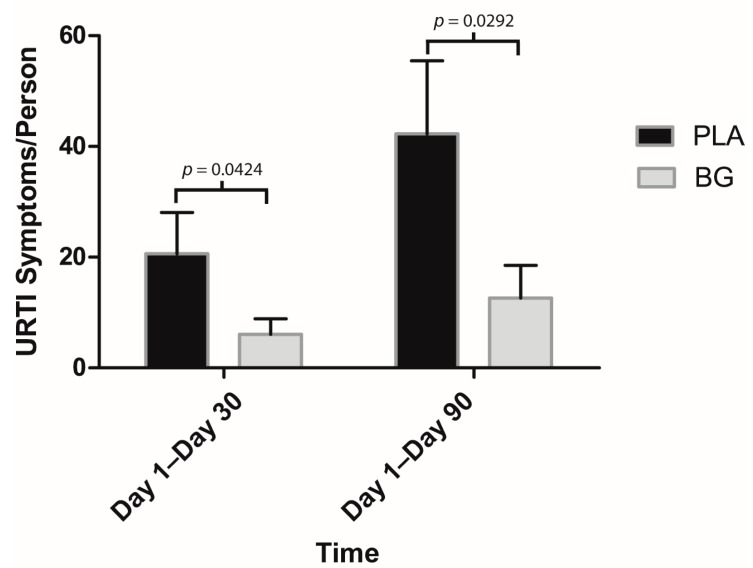
Number of URTI symptoms per person after 30 and 90 days of supplementation with BG or PLA in the PP population (*n* = 27). Values are mean ± SEM. BG, whole cell algae fermentate; PLA, placebo.

**Figure 5:**
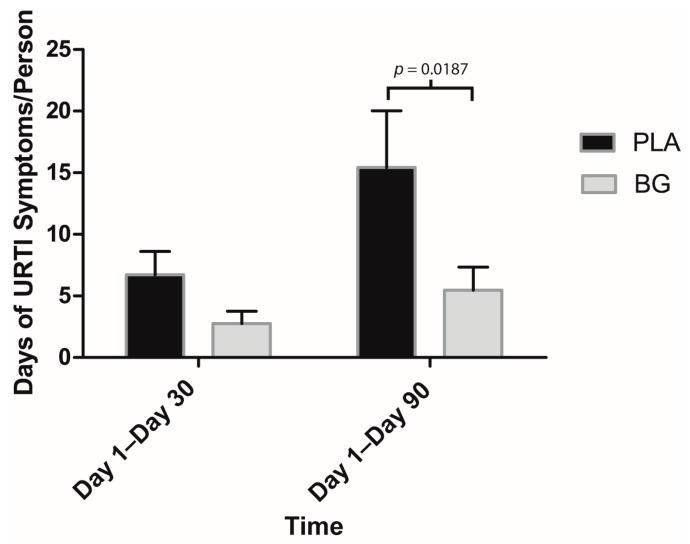
Mean number of days with at least one reported URTI symptom per person as assessed by the WURSS-24 daily questionnaire after 30 and 90 days of supplementation with BG or PLA in the PP population (*n* = 27). Values are mean ± SEM. BG, whole cell algae fermentate; PLA, placebo.

**Figure 6:**
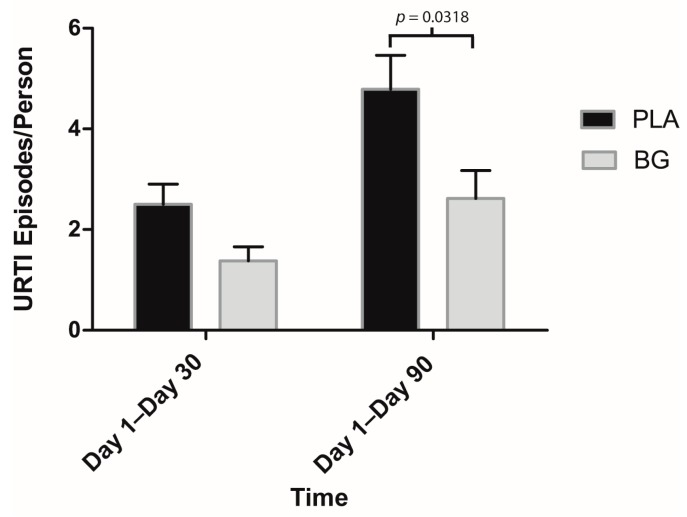
Mean number of URTI episodes per person after 30 and 90 days of supplementation with BG or PLA in the PP population (*n* = 27). Values are mean ± SEM. BG, whole cell algae fermentate; PLA, placebo.

**Figure 7:**
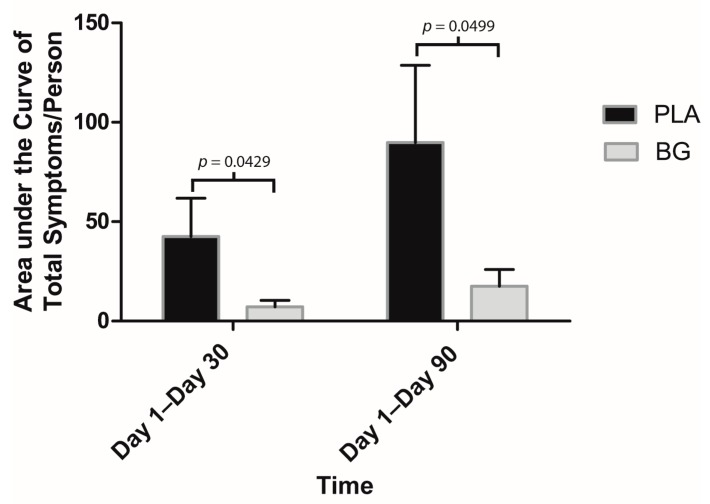
Mean area under the curve (AUC) for WURSS-24 daily symptoms after 30 and 90 days of supplementation with BG or PLA in the PP population (*n* = 27). Values are mean ± SEM. BG, whole cell algae fermentate; PLA, placebo.

Regarding secondary outcomes, participants in the BG group reported significantly fewer sick days compared to PLA from Day 1 to Day 30 (BG: 0.69 ± 0.55 days; PLA: 2.00 ± 0.83 days; *p* = 0.047) and Day 1 to Day 90 (BG: 1.46 ± 1.01 days; PLA: 4.79 ± 1.47 days; *p* = 0.041) (Figure 3). URTI symptoms per person were significantly lower in the BG group from Day 1 to Day 30 (BG: 6.08 ± 2.79; PLA: 20.64 ± 7.41; *p* = 0.042) and from Day 1 to Day 90 (BG: 12.62 ± 5.92; PLA: 42.29 ± 13.17; *p* = 0.029) than the PLA group (Figure 4). Symptom days per person were significantly lower in the BG group from Day 1 to Day 90 (BG: 5.46 ± 1.89 days; PLA: 15.43 ± 4.59 days; *p* = 0.019) than the PLA group (Figure 5). URTI episodes per person were significantly lower in the BG group from Day 1 to Day 90 (BG: 2.62 ± 0.67; PLA: 4.79 ± 0.67; *p* = 0.032) than the PLA group (Figure 6). Global severity was significantly lower in the BG group from Day 1 to Day 30 (BG: 7.19 ± 3.22; PLA: 42.54 ± 19.32; *p* = 0.043) and Day 1 to Day 90 (BG: 17.50 ± 8.41; PLA: 89.79 ± 38.92; *p* = 0.0499) compared to PLA (Figure 7).

**Figure 8:**
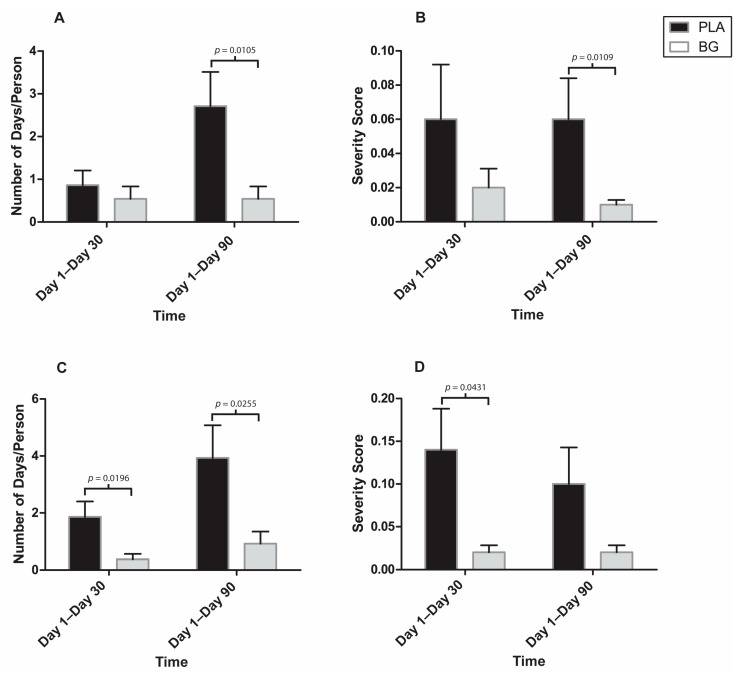
Individual WURSS-24 symptoms after 30 and 90 days of supplementation with BG or PLA in the PP population (*n* = 27) as assessed during post-hoc analysis. (**A**): Days with sore throat. (**B**): Severity score for sore throat. (**C**): Days with headache. (**D**): Severity score for headache. Values are mean ± SEM. BG, whole cell algae fermentate; PLA, placebo.

**Figure 9:**
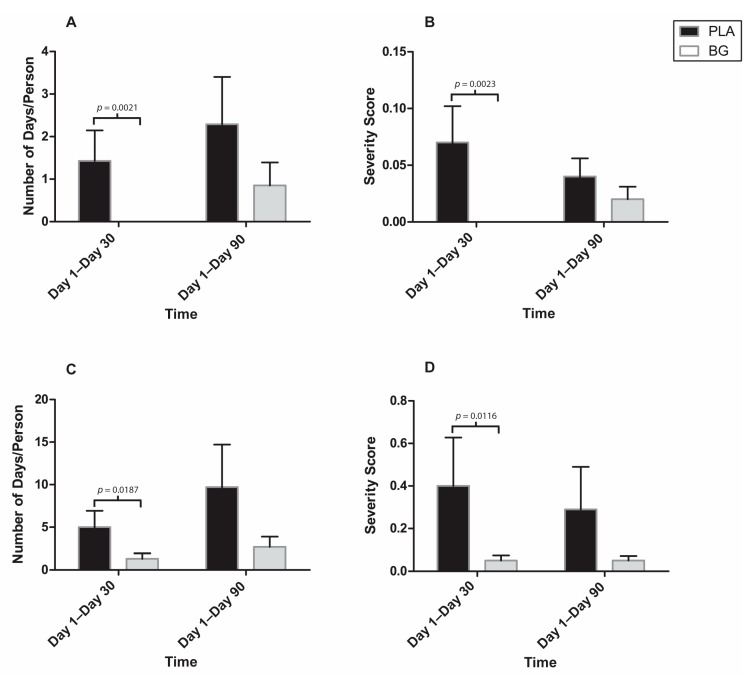
Individual WURSS-24 symptoms after 30 and 90 days of supplementation with BG or PLA in the PP population (*n* = 27) as assessed during post-hoc analysis: (**A**) days with body aches; (**B**) severity score for body aches; (**C**) days with symptom of feeling tired; and (**D**) severity score for symptom of feeling tired. Values are mean ± SEM. BG, whole cell algae fermentate; PLA, placebo.

**Figure 10:**
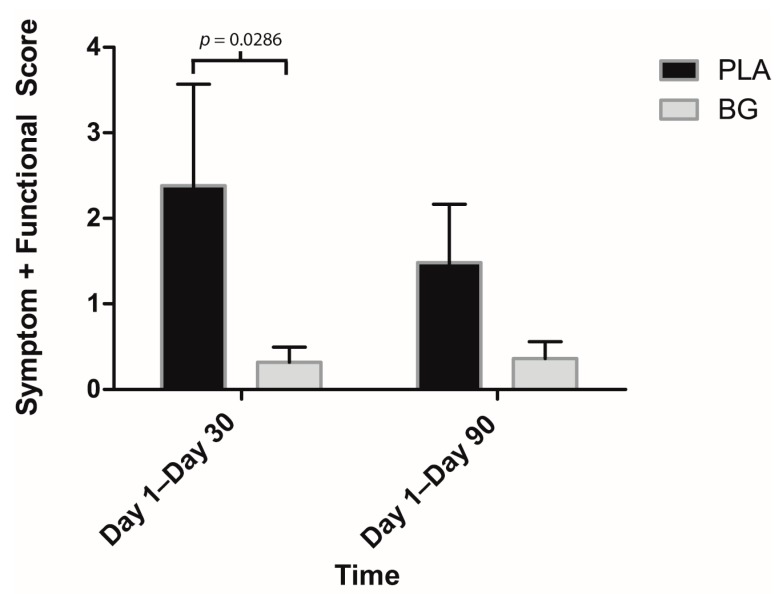
Score of symptoms plus functional outcomes from WURSS-24 after 30 and 90 days of supplementation with BG or PLA in the PP population (*n* = 27) as assessed during post-hoc analysis. Values are mean ± SEM. BG, whole cell algae fermentate; PLA, placebo.

The post-hoc analysis revealed that some individual symptoms were affected by supplementation with BG (Figure 8 and Figure 9). The duration of a sore throat was significantly lower in the BG group from Day 1 to Day 90 (BG: 0.54 ± 0.29 days; PLA: 2.71 ± 0.80 days; *p* = 0.011) compared to PLA. The severity of a sore throat was significantly lower in the BG group from Day 1 to Day 90 (BG: 0.01 ± 0.00; PLA: 0.06 ± 0.02; *p* = 0.011) compared to PLA. The duration of a headache was significantly lower in the BG group from Day 1 to Day 30 (BG: 0.38 ± 0.18 days; PLA: 1.86 ± 0.54 days; *p* = 0.020) and from Day 1 to Day 90 (BG: 0.92 ± 0.43; PLA: 3.93 ± 1.15; *p* = 0.026) compared to PLA. The severity of a headache was significantly lower in the BG group from Day 1 to Day 30 (BG: 0.02 ± 0.01; PLA: 0.14 ± 0.05; *p* = 0.043) compared to PLA. The duration of body aches was significantly lower in the BG group from Day 1 to Day 30 (BG: 0.00 ± 0.00 days; PLA: 1.43 ± 0.72 days; *p* = 0.002) compared to PLA. The severity of body aches was significantly lower in the BG group from Day 1 to Day 30 (BG: 0.00 ± 0.00; PLA: 0.07 ± 0.03; *p* = 0.002) compared to PLA. The duration of feeling tired was significantly lower in the BG group from Day 1 to Day 30 (BG: 1.31 ± 0.66 days; PLA: 5.00 ± 1.86 days; *p* = 0.0187) compared to PLA. The severity of feeling tired was significantly lower in the BG group from Day 1 to Day 90 (BG: 0.05 ± 0.02; PLA: 0.40 ± 0.22; *p* = 0.012) compared to PLA. Overall outcomes (symptom plus functional) are displayed in Figure 10. The overall outcomes were significantly lower in the BG group from Day 1 to Day 30 (BG: 0.32 ± 0.17; PLA: 2.38 ± 1.19 *p* = 0.029) compared to PLA.

Safety data are presented in Table 4 and Table 5. No significant differences were observed in vital signs or hematology (Table 5). In clinical chemistry measurements, potassium was significantly different between groups (BG: −0.01 ± 0.16 mmol/L; PLA: −0.27 ± 0.10 mmol/L; *p* = 0.004); however, the change in the PLA group was numerically larger than in the BG group, and all values were within the normal range for healthy adults. Therefore, this finding was considered clinically insignificant. The number of participants reporting AEs was not significantly different between groups. None of the AEs were categorized as “likely related” or “related” to the study products. No participants required medical treatment or hospitalization (i.e., no serious adverse events) and all AEs were resolved by the end of the study.

## 4. Discussion

To our knowledge, this is the first study to demonstrate significant benefits regarding URTI symptoms when supplementing with an algae-based fermentate in humans. Endurance-trained individuals supplemented with BG reported fewer sick days, URTI symptoms, URTI symptom days, URTI episodes, and lower global severity than those taking placebo. These findings suggest that incidence, duration, and severity of URTI symptoms were improved from BG supplementation over 90 days. Significant reductions in sick days, symptoms per person, and global severity were detected as early as the 30-day timepoint. Additionally, post-hoc analyses revealed benefits for specific symptoms, such as sore throat, headache, body aches, and feelings of tiredness. Post-hoc analyses showed improvement in overall outcomes via symptom plus functional outcome scores. Safety analyses measuring hematology, clinical chemistry, and vitals corroborated previous animal research [26], which confirmed BG to be safe and well-tolerated. No adverse events were reported that could be attributed to BG.

The novel findings in the current randomized, double-blind, placebo-controlled study on BG demonstrated efficacy in supporting immune health in endurance-trained individuals over a three-month period. Previous studies investigating URTI symptoms in similar populations have utilized marathons or intense exercise in the laboratory to stimulate transient immunosuppression over a shorter duration [26,31]. Talbott et al. found that for two weeks and four weeks post-marathon, fewer runners reported URTI symptoms when supplementing with a yeast-derived β-glucan supplement [32]. In a similar study, supplementing with yeast-derived β-glucan for 28 days post-marathon resulted in fewer URTI symptom days [31]. Importantly, the present study extended previous findings under conditions of acute, exercise-induced immunosuppression by demonstrating benefits over a longer duration in exercising individuals who may be at higher risk for URTI due to their typical exercise regimen. Previous studies conducted with yeast-derived β-glucan supplementation evaluating URTI over a 90-day period examined older adults and stressed women, who may also be at higher risk for developing URTI [33,34]. Therefore, the present study was the first study to investigate the efficacy of a β-glucan product in endurance-trained individuals over a three-month period.

Post-hoc evaluation of specific symptoms and quality of life outcomes revealed that sore throat, headache, body aches, and feelings of tiredness were significantly lower in duration and severity in BG compared to PLA. These data are consistent with the literature following β-glucan consumption. A double-blind, randomized, placebo-controlled parallel study found severity ratings for sore throat were lower post-marathon when supplemented with β-glucan [35]. While feeling tired is considered a cold symptom on the WURSS-24, it also affects daily functioning. Additionally, improvements were observed with BG in symptom plus functional outcome scores. Feldman et al. showed that participants reported a higher quality of life after consuming β-glucan for 12 weeks of cold and flu season [36]. These data suggest that BG may have a positive impact on certain aspects of daily functioning and quality of life in endurance-trained individuals.

The findings from this study are not surprising given the significant amount of research linking insoluble β-(1,3)-glucan (with and without β-(1,6)-linkages) from various sources with human immunity [12,21,37,38]. β-(1,3)-glucans are recognized by specific pathogen recognition receptors on immune cells including dectin-1, toll-like receptors (TLRs), complement receptor 3 (CR3), scavenger receptor, and lactosylceramide [39,40,41,42,43]. Notably, the main receptor for β-(1,3)-glucan is dectin-1 [39,40,43,44]. Recognition of β-(1,3)-glucan by the dectin-1 receptor triggers an innate immune response that mediates protection against microbes [43,45]. Importantly, while dectin-1 can bind both soluble and particulate/insoluble β-(1,3)-glucan, only particulate β-glucan, such as the BG in this study, appears capable of activating dectin-1 signaling to initiate an immune response [45]. The present findings indicate that BG supports the immune system in a clinically relevant manner by maintaining health that was noted through reduced incidence, duration, and severity of URTI symptoms, which were all observed in the current study.

Some limitations exist regarding the current study. Due to the inclusion and exclusion criteria, there were challenges in recruitment. To control for seasonal variations in URTI incidence and lifestyle behaviors such as exercise participation and dietary intake, the enrollment period was terminated before the *a priori* defined number of participants were recruited. Nonetheless, statistical significance was still achieved for multiple outcomes from the WURSS-24 and these outcomes were aligned with and supported by the literature on yeast β-glucan ingredients supplemented for 90 days or greater [33,34,46]. This was also the likely reason there were no statistically significant findings in the primary outcome, although trends were observed in very mild symptoms. In addition, the primary outcome was based on a previous study measuring symptoms in a similar population consuming a β-glucan supplement where suppression of the immune system was initiated by a marathon, whereas the current study tested over a longer duration without utilizing a single bout of exercise for immunosuppression [32]. Therefore, an outcome such as sick days or global severity may be a more sensitive measurement in the current study design.

## 5. Conclusions

In conclusion, the current study showed the ability of a proprietary *Euglena gracilis* fermentate with >50% β-(1,3)-glucan and >15% protein to support the immune system with a significant reduction in self-reported sick days, total number of symptoms, number of days with symptoms, number of URTI episodes, and global severity of symptoms in endurance-trained individuals. As a novel source of algal β-(1,3)-glucan, BG derived from a proprietary strain of *Euglena gracilis* might be a valuable addition to nutritional supplementation. It is beneficial by proactively lessening the burden of URTI symptoms in individuals whose diets and lifestyles do not support optimal immune function and health. The effect of nutritional supplements on the immune response is a burgeoning area for researchers, and BG supplementation presents a promising strategy to support a healthy immune system.

## Figures and Tables

**Table 1:** Inclusion and exclusion criteria.

**Inclusion Criteria**
1. Males and females 21 to 65 years of age
2. BMI > 18 kg/m^2^ to <35 kg/m^2^
3. Wash-out period for supplements affecting immune function
4. Females agree to use of appropriate birth control
5. Consistent diet and lifestyle routine
6. Abstain from exercising, tobacco use, and supplements at study visits
7. Abstain from music, computer/cell phone use at study visits
8. Abstain from consuming candy, chewing gum at study visits
9. Abstain from caffeine for 1 h prior to and at study visits
10. Participated in an endurance exercise for 1.5–3 h/day for 5–6 days per week
11. Healthy as determined by laboratory results and medical history
12. Willingness to perform study procedures and to complete all clinic visits.
13. Has given voluntary, written, informed consent
**Exclusion Criteria**
1. Women who were pregnant or breastfeeding
2. Previous major gastrointestinal surgery or digestive disorder a. inflammatory bowel disease, irritable bowel syndrome, chronic constipation, and history of chronic diarrhea; history of surgery for weight loss, gastroparesis, or clinically important lactose intolerance; and chronic GI illness.
3. Consumed beta-glucan supplements and unwilling to wash out for 4 weeks
4. Consumption of anti-inflammatory medications (81 mg aspirin is acceptable)
5. Upper respiratory tract infection at baseline
6. Antibiotics within 4 weeks of screening
7. Chronic inflammatory condition
8. Type I or Type II diabetes or clinically important renal, hepatic, cardiac, pulmonary, pancreatic, neurologic, or biliary disorder, or a recent history of cancer other than non-melanoma skin cancer.
9. Current use of antipsychotic medications
10. Allergies, chronic bronchitis, asthma, or wheezing
11. Auto-immune disorders
12. Unusual sleep routine
13. Use of immunomodulators
14. Consumed supplements affecting immune function
15. Chronic use of Antacids and Proton Pump Inhibitors
16. Prebiotics and Probiotics
17. Unwilling to have blood drawn
18. Diagnosed depression in the 2 years prior to screening
19. Eating disorders or extreme dietary habits
20. Use of marijuana
21. Active infection or signs/symptoms of an acute infection at study visits.
22. Heavy use of tobacco
23. Consumption of ≥ 14 drinks per week
24. Alcohol or drug abuse within the last 2 years
25. Blood donation during the study or within 30 days of completing the study
26. Unwilling to comply with study procedures and study product consumption
27. Known allergy to the test material’s active or inactive ingredients
28. Unstable medical conditions as assessed by the QI
29. Clinically significant abnormal laboratory results at screening
30. Participation in a clinical research trial within 30 days prior to randomization

**Table 2:** Demographic characteristics of participants with whole cell algae fermentate (BG) and placebo (PLA) in the PP population (*n* = 27).

Parameter	BG *n* = 13	PLA *n* = 14	Between Group *p*-Value *
Age (years)			
Mean +/− SEM	46.77 ± 3.67 (13)	42.93 ± 3.29 (14)	0.4346
Gender (*n* (%))			0.8632
Female	7 (53.8%)	8 (57.1%)	
Male	6 (46.2%)	6 (42.9%)	
Ethnicity (*n* (%))			
Eastern European White	2 (15.4%)	1 (7.1%)	0.3933
South Asian	1 (7.7%)	0 (0%)	
Western European White	10 (76.9%)	13 (92.9%)	

*n*, number; SEM, standard error of the mean; * For continuous parameters, between group *p*-values were generated from ANOVA models with Group as a fixed effect. For categorical parameters, between group *p*-values generated by Chi-square or Fisher’s Exact (2-tail) tests as appropriate.

**Table 3:** Percentage (%) of participants with URTI symptoms from Day 1 to Day 30, and from Day 1 to Day 90 of supplementation with whole cell algae fermentate (BG) or placebo (PLA) in the PP population (*n* = 27).

Symptom	Interval	BG *n* = 13 *n* (%)	PLA *n* = 14 *n* (%)	Between Group *p*-Value *
Very Mild	Days 1–30	53.8% (7)	85.7% (12)	0.1032
	Days 1–90	76.9% (10)	100.0% (14)	0.0978
Mild	Days 1–30	30.8% (4)	57.1% (8)	0.2519
	Days 1–90	46.2% (6)	64.3% (9)	0.4495
Moderate	Days 1–30	0.0% (0)	21.4% (3)	0.2222
	Days 1–90	15.4% (2)	28.6% (4)	0.6483
Severe	Days 1–30	7.7% (1)	14.3% (2)	1.0000
	Days 1–90	15.4% (2)	14.3% (2)	1.0000
Any	Days 1–30	69.2% (9)	85.7% (12)	0.3845
	Days 1–90	92.3% (12)	100.0% (14)	0.4815

*n*, number; %, percent; * Between group *p*-value generated by Fisher’s Exact (2-tail) test.

**Table 4:** Clinical chemistry parameters measured in participants in the safety population with whole cell algae fermentate (BG) or placebo (PG) at screening (Visit 1) and Day 90 (Visit 3) (*n* = 34).

Parameter	Study Day	BG *n* = 17 Mean ± SEM (*n*)	PLA *n* = 17 Mean ± SEM (*n*)	Between Group *p*-Value *	Reference Value
Creatinine (µmol/L)	Screening	82.82 ± 4.43 (17)	80.00 ± 3.41 (17)	0.6169	44–97
	Day 90	78.81 ± 4.32 (16)	79.59 ± 3.26 (17)		
	Change from Screening to Day 90	−2.69 ± 1.82 (16)	−0.41 ± 1.33 (17)	0.3510	
eGFR (mL/min/1.73)	Screening	86.59 ± 4.05 (17)	89.24 ± 3.33 (17)	0.6172	60–120
	Day 90	89.56 ± 4.51 (16)	87.24 ± 3.18 (17)		
	Change from Screening to Day 90	2.25 ± 1.91 (16)	−2.00 ± 1.57 (17)	0.1075	
Sodium (mmol/L)	Screening	142.00 ± 0.57 (17)	142.35 ± 0.50 (17)	0.6442	133–148
	Day 90	142.06 ± 0.66 (16)	141.76 ± 0.41 (17)		
	Change from Screening to Day 90	0.19 ± 0.73 (16)	−0.59 ± 0.56 (17)	0.5635	
Potassium (mmol/L)	Screening	4.79 ± 0.09 (17)	4.62 ± 0.13 (15)	0.2944	3.3–5.7
	Day 90	4.81 ± 0.09 (16)	4.36 ± 0.08 (17)		
	Change from Screening to Day 90	−0.01 ± 0.16 (16)	−0.27 ± 0.10 (15)	0.0038	
Chloride (mmol/L)	Screening	101.88 ± 0.62 (17)	102.82 ± 0.46 (17)	0.2319	98–115
	Day 90	101.56 ± 0.57 (16)	102.35 ± 0.36 (17)		
	Change from Screening to Day 90	−0.31 ± 0.77 (16)	−0.47 ± 0.39 (17)	0.4314	
Total Bilirubin (µmol/L)	Screening	7.64 ± 0.92 (17)	10.00 ± 1.67 (17)	0.5192 (*r*)	≤25
	Day 90	9.31 ± 4.67 (16)	11.24 ± 1.65 (17)		
	Change from Screening to Day 90	1.57 ± 1.14 (16)	1.24 ± 0.68 (17)	0.9531	
Aspartate Transaminase (AST) (U/L)	Screening	25.41 ± 1.62 (17)	19.40 ± 1.32 (15)	0.0081	7–70
	Day 90	21.75 ± 1.82 (16)	28.18 ± 9.01 (17)		
	Change from Screening to Day 90	−3.19 ± 1.90 (16)	9.87 ± 9.47 (15)	0.1107 (*r*)	
Alanine Transaminase (ALT) (U/L)	Screening	25.82 ± 0.77 (17)	17.65 ± 1.18 (17)	0.0246 (*r*)	12–90
	Day 90	19.49 ± 2.27 (16)	20.76 ± 2.64 (17)		
	Change from Screening to Day 90	−3.76 ± 2.06 (16)	3.12 ± 2.35 (17)	0.0990 (*r*)	
Calcium (mmol/L)	Screening	2.36 ± 0.01 (17)	2.35 ± 0.02 (17)	0.9277	1.8–3.0
	Day 90	2.41 ± 0.02 (16)	2.38 ± 0.02 (17)		
	Change from Screening to Day 90	0.06 ± 0.015 (16)	0.02 ± 0.02 (17)	0.1735	

*n*, number; SEM, standard error of the mean; + within group *p*-values generated by the Paired *t*-test or the Wilcoxon Signed Rank test. (*r*) indicates Wilcoxon; * for Baseline (Day 0), between group *p*-value generated by ANOVA with Group as a fixed effect. Between group *p*-value for the Change from Baseline was generated from ANCOVA with baseline as a covariate and Group as a fixed effect. (*r*) indicates values were ranked prior to generating ANOVA or ANCOVA.

**Table 5:** Hematology parameters measured in participants in the safety population with whole cell algae fermentate (BG) or placebo (PLA) at screening (Visit 1) and Day 90 (Visit 3) (*n* = 34).

Parameter	Study Day	BG *n* = 17 Mean ± SEM (*n*)	PLA *n* = 17 Mean ± SEM (*n*)	Between Group *p*-Value *	Reference Value
Hemoglobin (g/L)	Screening	134.94 ± 2.54 (17)	139.53 ± 2.96 (17)	0.2482	135–175 (M), 120–160 (F)
	Day 90	137.75 ± 2.12 (16)	141.12 ± 2.66 (17)		
	Change from Screening to Day 90	4.13 ± 1.97 (16)	1.59 ± 1.59 (17)	0.7404	
Hematocrit (L/L)	Screening	0.40 ± 0.007 (17)	0.42 ± 0.009 (17)	0.1809	0.4–0.5 (M), 0.35–0.45 (F)
	Day 90	0.41 ± 0.005 (16)	0.42 ± 0.007 (17)		
	Change from Screening to Day 90	0.01 ± 0.005 (16)	0.01 ± 0.005 (17)	0.9021	
White Blood Cell Count (×10^9^/L)	Screening	5.91 ± 0.33 (17)	5.59 ± 0.30 (17)	0.4787	4.0–10.0
	Day 90	5.44 ± 0.29 (16)	5.12 ± 0.31 (17)		
	Change from Screening to Day 90	−0.49 ± 0.28 (16)	−0.48 ± 0.27 (17)	0.6987	
Red Blood Cell Count (×10^12^/L)	Screening	4.50 ± 0.09 (17)	4.69 ± 0.10 (17)	0.1797	4.5–6.0 (M), 4.0–5.1 (F)
	Day 90	4.62 ± 0.065 (16)	4.72 ± 0.09 (17)		
	Change from Screening to Day 90	0.13 ± 0.065 (16)	0.04 ± 0.053 (17)	0.7215	
MCV (fl)	Screening	89.53 ± 0.71 (17)	89.59 ± 1.02 (17)	0.9626	80–100
	Day 90	89.19 ± 0.79 (16)	89.82 ± 0.96 (17)		
	Change from Screening to Day 90	−0.06 ± 0.335 (16)	0.24 ± 0.36 (17)	0.5145	
MCH (pg)	Screening	30.01 ± 0.35 (17)	29.81 ± 0.33 (17)	0.6814	27.5–33.0
	Day 90	29.83 ± 0.28 (16)	29.90 ± 0.34 (17)		
	Change from Screening to Day 90	0.01 ± 0.14 (16)	0.09 ± 0.01 (17)	0.5950	
MCHC (g/L)	Screening	334.71 ± 2.07 (17)	333.59 ± 1.92 (17)	0.6945	305–360
	Day 90	334.38 ± 1.58 (16)	333.29 ± 1.67 (17)		
	Change from Screening to Day 90	0.63 ± 1.85 (16)	−0.29 ± 1.71 (17)	0.6190	
RDW (%)	Screening	13.44 ± 0.59 (17)	13.61 ± 0.56 (17)	0.4088	11.5–14.5
	Day 90	13.41 ± 0.80 (16)	13.58 ± 0.54 (17)		
	Change from Screening to Day 90	−0.01 ± 0.15 (16)	−0.03 ± 0.075 (17)	0.3380 (*r*)	
Platelets (×10^9^/L)	Screening	262.53 ± 14.94 (17)	243.18 ± 11.69 (17)	0.3153	150–00
	Day 90	244.63 ± 11.70 (16)	242.71 ± 13.08 (17)		
	Change from Screening to Day 90	−21.94 ± 11.56 (16)	−0.47 ± 6.76 (17)	0.2571	
Absolute: Neutrophils (×10^9^/L)	Screening	3.28 ± 0.27 (17)	3.29 ± 0.24 (17)	0.9738	2.0–7.5
	Day 90	3.07 ± 0.24 (16)	2.93 ± 0.24 (17)		
	Change from Screening to Day 90	−0.23 ± 0.26 (16)	−0.36 ± 0.22 (17)	0.6352	
Absolute: Lymphocytes (×10^9^/L)	Screening	1.96 ± 0.12 (17)	1.73 ± 0.09 (17)	0.1303	1.0–3.5
	Day 90	1.78 ± 0.11 (16)	1.63 ± 0.11 (17)		
	Change from Screening to Day 90	−0.20 ± 0.01 (16)	−0.10 ± 0.08 (17)	0.8927	
Absolute: Monocytes (×10^9^/L)	Screening	0.52 ± 0.05 (17)	0.41 ± 0.03 (17)	0.1665 (*r*)	0.2–1.0
	Day 90	0.44 ± 0.04 (16)	0.41 ± 0.03 (17)		
	Change from Screening to Day 90	−0.07 ± 0.04 (16)	−0.01 ± 0.03 (17)	0.7287	
Absolute: Eosinophils (×10^9^/L)	Screening	0.15 ± 0.03 (17)	0.14 ± 0.02 (17)	1.0000 (*r*)	0.0–0.5
	Day 90	0.13 ± 0.02 (16)	0.13 ± 0.02 (17)		
	Change from Screening to Day 90	−0.01 ± 0.02 (16)	−0.01 ± 0.02 (17)	0.7022 (*r*)	
Absolute: Basophils (×10^9^/L)	Screening	0.01 ± 0.007 (17)	0.01 ± 0.004 (17)	0.5595 (*r*)	0.0–0.2
	Day 90	0.01 ± 0.008 (16)	0.01 ± 0.004 (17)		
	Change from Screening to Day 90	0.00 ± 0.01 (16)	0.00 ± 0.01 (17)	0.4850 (*r*)	

*n*, number; SEM, standard error of the mean; Min, minimum; Max, maximum; M, males; F females; + within group *p*-values generated by the Paired t-test or the Wilcoxon Signed Rank test. (*r*) indicates Wilcoxon; * for Baseline (Day 0), between group *p*-value generated by ANOVA with Group as a fixed effect; * Between group *p*-value for the Change from Baseline was generated from ANCOVA with baseline as a covariate and Group as a fixed effect. (*r*) indicates values were ranked prior to generating ANOVA or ANCOVA.

## Supplementary Materials

The following are available online at https://www.mdpi.com/2072-6643/11/12/2926/s1, Table S1: CONSORT 2010 checklist of information to include when reporting a randomised trial.
